# Enhancing Positioning Accuracy in Urban Terrain by Fusing Data from a GPS Receiver, Inertial Sensors, Stereo-Camera and Digital Maps for Pedestrian Navigation

**DOI:** 10.3390/s120606764

**Published:** 2012-05-25

**Authors:** Baranski Przemyslaw, Strumillo Pawel

**Affiliations:** Institute of Electronics, Technical University of Lodz, Wolczanska 211/215, 90-924 Lodz, Poland; E-Mail: pawel.strumillo@p.lodz.pl

**Keywords:** particle filtering, stereovision, digital maps, GPS, Monte Carlo, dead reckoning

## Abstract

The paper presents an algorithm for estimating a pedestrian location in an urban environment. The algorithm is based on the particle filter and uses different data sources: a GPS receiver, inertial sensors, probability maps and a stereo camera. Inertial sensors are used to estimate a relative displacement of a pedestrian. A gyroscope estimates a change in the heading direction. An accelerometer is used to count a pedestrian's steps and their lengths. The so-called probability maps help to limit GPS inaccuracy by imposing constraints on pedestrian kinematics, e.g., it is assumed that a pedestrian cannot cross buildings, fences *etc*. This limits position inaccuracy to *ca.* 10 m. Incorporation of depth estimates derived from a stereo camera that are compared to the 3D model of an environment has enabled further reduction of positioning errors. As a result, for 90% of the time, the algorithm is able to estimate a pedestrian location with an error smaller than 2 m, compared to an error of 6.5 m for a navigation based solely on GPS.

## 1. Introduction

GPS-NAVSTAR (Global Positioning System-NAVigation Signal Timing And Ranging), popularly known as the GPS system, has considerably gained in civilian interests, since May 2000. Earlier, the system was practically reserved for military purposes. The positioning accuracy for the civilian sector was *ca.* 100 m due to an intentional error, called Selective Availability. According to report [1], the horizontal positioning error is less than 17 m for 99% of the time in average conditions or 17 m for 90% of the time in worse outdoor conditions. The error depends on many factors, like atmospheric conditions, sun activity, geographical location, terrain type, satellites' constellation, etc. In an open space, positioning errors are of *ca.* 2–3 m. However, in dense built-up areas, the location error may reach 100 m [2,3] or even more [4]. The error is introduced due to multipath propagation of signals transmitted by the satellites when there is no line-of-sight. A satellite signal is bounced off the walls of a building before finding its way to a GPS receiver. The propagation time of the signal is delayed and the GPS receiver miscalculates its location with a reference to the satellites.

There are many techniques to improve the location accuracy. Along coasts, for marine purposes, special ground DGPS (Differential GPS) reference stations broadcast differential corrections that allow a GPS receiver to eliminate tropospheric, ionospheric, ephemeris and clock errors. The overall error is reduced to 10 m with accuracy decreasing by 1 m with each 150 km increase in distance from the reference station. The corrections are transmitted on a *ca.* 300 kHz carrier frequency. A receiver must be, however, equipped with an additional antenna [5].

A-GPS (Assisted GPS) is a technique that downloads from the Internet the data concerning GPS satellite constellation [6]. Otherwise a GPS receiver may require up to 12.5 minutes to receive data about satellite constellation. By employing the A-GPS, the first fix is provided within few seconds. Additionally, a GSM modem can support a GPS receiver with a rough location which is obtained by measuring the strengths of signals from GSM base stations.

Apart from L1 = 1,227 MHz, a second frequency called L2C = 1,575 MHz, has been made available to the civilian sector with the aim of reducing the ionospheric and tropospheric errors which are a well defined function of frequency. Also, the so-called augmentation systems effectively reduce tropospheric and ionospheric errors by sending differential corrections from geostationary satellites directly to GPS receivers. The geostationary satellites imitate also GPS satellites and improve mainly the vertical accuracy. EGNOS (European Geostationary Navigation Overlay Service) works in Europe, WAAS (Wide Area Augmentation System) in the US, MSAS (Multi-functional Satellite Augmentation System) in Japan and GAGAN (GPS aided Geo-Augmented Navigation) in India.

Real Time Kinematics technique achieves the accuracy of millimetres in an open space and is designated for cartographic measurements. However, two GPS receiver are necessary. The base receiver is placed in a known position and calculates tropospheric, ionospheric, ephemerids and clock errors of satellites. The corrections are sent via a radio link to a mobile GPS receiver. In the trials reported in [4] RMS error of 2 cm on a mountain high-way was noted whereas trials in urban canyons yielded a 50 m RMS error.

All the above mentioned techniques are helpless against multipath propagation errors. Algorithms harnessed in car navigation alleviate these errors by taking advantage of car kinematics and a comparatively sparse network of roads, as described, e.g., in [7] or [8]. An error of 20 m is of no bigger importance to a driver. In case of navigating pedestrians, especially blind ones, the target accuracy should ideally not exceed the pavement width, *i.e., ca.* 2 m.

This work presents a navigation scheme using GPS readouts, digital maps, inertial sensors and stereovision images with an aim of navigating a blind pedestrian. An accelerometer is used to detect pedestrian's strides and estimate their length. A gyroscope serves for estimating the heading direction. Those data sources help eliminate gross positioning errors and outliers. The digital maps are used twofold. Firstly, the so-called probability map is built to eliminate improbable user transitions, like traversing water ponds, crossing walls and buildings, etc. Secondly, a 3D model of the environment is built. The model is compared to stereoscopic images recorded by a mobile stereo camera. Interestingly, this comparison of 3D geometry of the environment provides good positioning accuracy in the surrounding of buildings, where GPS readouts are compromised. The proposed scheme employs the particle filter, also known as a sequential Monte Carlo method.

## 2. Related Work

The topic of pedestrian navigation including navigation aids for blind pedestrians has been described in many publications [9–11]. The first step to correct GPS readouts is to apply inertial sensors. Inertial sensors are mainly used in aviation to compute the orientation and position of an aircraft. Calculating the location requires double integration of the acceleration vector. Prior to these calculations the gravity acceleration must be removed, as accelerometers cannot distinguish gravity from an aircraft's accelerations. Therefore, the orientation of an aircraft with respect to the Earth's surface must be calculated from gyroscopes. The technique is known as INS (Inertial Navigation Systems) and warrants very precise and expensive laser sensors using the Sagnac effect. However, the strict implementation of INS using MEMS sensors (Micro Electro-Mechanical Systems) is useless after few seconds due to errors growing quadratically with time [12].

A travelled distance of a pedestrian can be estimated with surprisingly good accuracy by measuring the length of steps. This is done by analysing the acceleration in the gravity axis [10,13]. As a person walks, the body undulates according to the strides. The technique is accurate from 0.5% to 10%, depending on the gait style. ZUPT (Zero Velocity Update) technique exploits the fact that a foot is at rest for some short period. An accelerometer must be mounted to a foot which is an inconvenience, offset however by better accuracy compared to the previous method [14].

A heading direction can simply be read out from a magnetic compass, optionally supported by a gyroscope. A compass is sensitive to local distortions of a magnetic field due to cars, power lines *etc.* [10]. An electric tram can compromise a compass readouts within the radius of up to 100 m. A gyroscope, coupled by the Kalman filter, can reduce erroneous readouts [15].

The combination of GPS and inertial sensors readouts provides continuous estimates during GPS outages in harsh environments like tunnels, underground passages, dense urban areas etc. Positioning data from these two sources are usually integrated by the Extended Kalman filter or particle filter, which perform well when errors can be modelled by white noise which has the property of being uncorrelated with itself. However, the GPS errors are characterized by coloured noise [2,16]. This is because when a GPS receiver loses track of satellites its position is estimated by using the history of previous locations. Secondly, signals from satellites occluded by the same building are equally delayed, which introduces a bias in a given direction. This feature of errors corrupting GPS readouts was reported in earlier studies [14,17]. Jirawimut *et al.* [18] present an interesting concept where the height of buildings wereutilized to check if a given satellite is occluded. The authors carried out simulation which yielded good results.

To improve the accuracy of dead-reckoning techniques, an additional source of the absolute pedestrian location should be introduced. Experiments in [4] showed that a combination of GPS and GLONASS navigation systems reduces root-mean-square (RMS) error by half and outliers by several times. Another solution is introducing the so-called probability map as a new source of data, which defines regions of a terrain that the user is most likely to enter or cross. A pedestrian is more likely to walk along pavements, parking places, paths, *etc.*, rather than crossing walls, water ponds, *etc*. This cuts down the positioning errors as they start to aggregate. This map-based navigation concept proved its usefulness in outdoor pedestrian applications [19,20] as well as indoor positioning [21–23]. In built-up areas, a probability map limits inaccuracy to a width of an urban canyon, usually delineated by buildings on both sides.

A further improvement of positioning accuracy requires positioning with respect to known landmarks, *i.e.*, the so-called exteroception. The accuracy of landmarks' location should be approximately an order of magnitude better than the target accuracy of *ca.* 2 m. A vision-based technique called SLAM (Simultaneous Localization and Mapping) enables to build a map of the surrounding space and provide localization at the same time without employing any global navigation method. The SLAM based technique can be applied to build a 3D map of the environment. A review of SLAM techniques, modifications, results, *etc.*, are given in book [24]. An application of SLAM for pedestrian navigation with results is given in [25]. Also a navigation system for the blind using a stereo-camera for tracing landmarks is presented in [26].

On the other hand, precise maps of outdoor or indoor environments may already be available, e.g., a plan of a building or plans of a city which are accurate to single centimetres, accuracy hardly achievable by any SLAM techniques. There are different sensors used to compare the environmental map with a robot's or pedestrian's location. Ultrasound sensors measure distance with 1% accuracy, being at the same time very cheap and compact. The maximum achievable distance is *ca.* 10 m. The angular resolution, however, is poor, ±15°. Laser scanners provide 0.1% distance measurement accuracy with a maximum distance of *ca.* 50 m. The angular resolution is of a fraction of a degree. The scanning is omnidirectional. The power consumption is *ca.* 10 W for longer distances. The size of a laser sensor is around 5 cm by 5 cm by 5 cm. The cost is rather high (*ca.* $4,000 USD depending on accuracy, maximum distance *etc.*). These sensors are commonly used in robotics. Time-of-Flight (ToF) cameras offer similar parameters as laser sensors providing 3D reconstruction of the scanned environment. The angle of view is *ca.* 30°. Application of laser scanners and ToF cameras is limited to indoor environments due sunlight interference.

In [27] ultrasound and laser sensing techniques are compared for indoor positioning of a robot. Work [23] presents an indoor localization system based on the particle filter. The user wears ultrasound sensors which measure a distance to adjacent walls. Therefore, the user location can be compared against the alignment of building walls. Application of an ultrasound sensor improved the positioning accuracy by 7 times in good trial conditions, *i.e.*, with no additional objects in the building's corridor, closed doors *etc*. A similar technique using a laser sensor mounted on a white cane is reported in [22]. A laser sensor detects corners of corridors what provides a comparison with a building's plan.

Finally, stereovision is a passive imaging technique that can be used indoors and outdoors. The cameras can be compact and inexpensive. However, stereovision imaging is limited to the environments featuring good lighting conditions, requires calibration of the stereovision optics and offers worse depth estimation accuracy than the earlier outlined active methods [28]. Moreover, demanding computations are required for calculating depth maps. In stereovision, depth accuracy can range from a few centimetres for objects located in a few metres range to a few metres for more distant objects. The so-called subpixel interpolation methods were proposed to improve depth estimation accuracy [29].

The work presented here combines global and local positioning techniques. GPS location estimates are augmented by dead reckoning techniques derived from inertial sensors, *i.e.*, gyroscopes and accelerometers. A stereo-camera is used for distance measurements. Finally, a digital map of the outdoor terrain is incorporated into the algorithm. Each of the listed source of positioning data features different error characteristic, e.g., in open spaces GPS readouts are most accurate whereas stereovision may yield large depth estimation errors. Conversely, for city canyons where GPS accuracy falters, stereovision and the digital map offer good positioning. A particle filtering algorithm is proposed to optimally fuse all data source.

## 3. A Prototype System for Pedestrian Positioning

**Figure 1.**
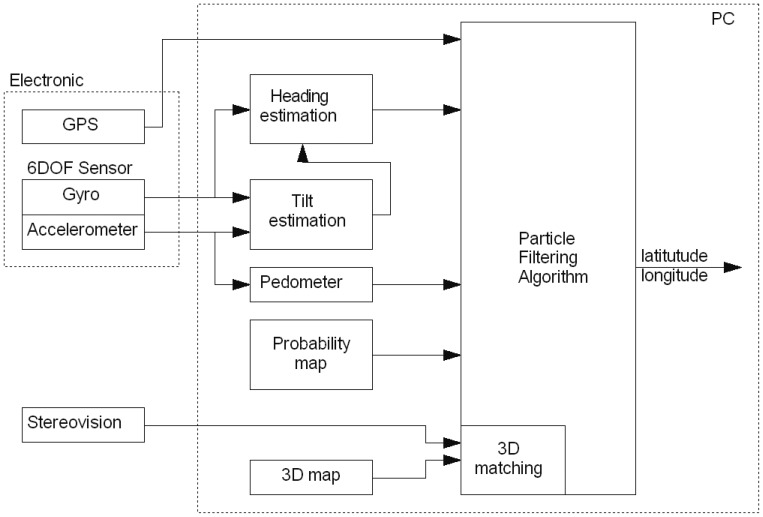
The block diagram of the system. The electronic module is a dedicated electronic circuit comprised of a 6DOF sensor, GPS receiver and microcontroller. The module is connected with the PC via USB interface. The stereovision block represents a stereo camera.

A block diagram of the proposed system is presented in Figure 1. From the hardware point of view, the system is made up of three parts: a PC platform, stereo camera connected through FireWire interface and an electronic module housing a GPS receiver and 6DOF sensor. The data was acquired by walking through the University Campus with the stereovision camera attached to the chest of an experimenter. The laptop stored data from the electronic module and stereo camera. Then the data was processed off-line on a PC.

### 3.1. Electronic Module

**Figure 2.**
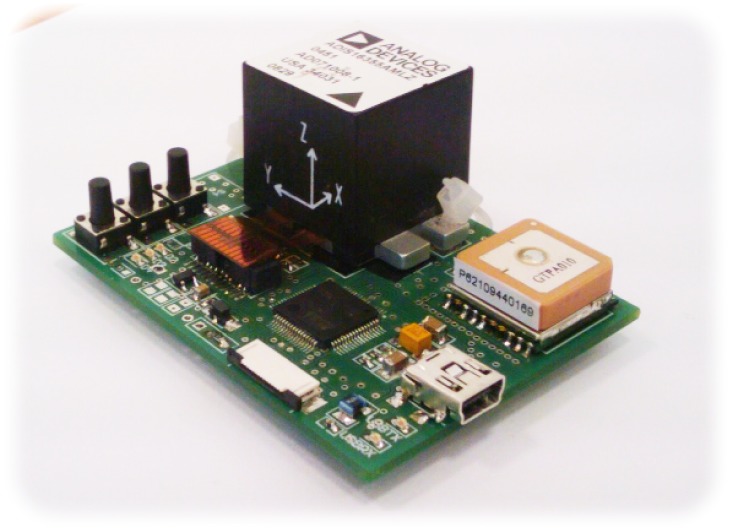
A picture of the built electronic module. The black box is a 6DOF sensor—3-axial gyroscope and 3-axial accelerometer. The orange cuboid is an antenna of a GPS receiver. The module is connected with the PC through a mini-USB socket.

The built electronic module, see Figure 2, is a dedicated PCB with a microcontroller, 6DOF sensor and GPS receiver. The microcontroller reads out data from the 6DOF sensor, ADIS16355 at a rate of 820 Hz. Downsampling is carried out to limit data stream to the PC. An 8th order, lowpass Chebyshev filter is used to avoid aliasing. Samples are sent to the computer at a rate of 205 Hz. The module sends data through the USB interface.

#### 3.1.1. 6DOF Sensor

The 6DOF sensor, ADIS16355 from Analog Devices, comprises a 3-axial accelerometer and 3-axial gyro. The data from the sensor is read out through a digital interface, SPI. The samples are of 14-bit resolution whereby practically the three least significant bits are random. The ADIS16355 has been superseded by much less noisy ADIS16375. The static parameters of the former were investigated by the Allan variance [30].

##### Gyroscope

The gyroscope is used to estimate the pedestrian orientation. Angular velocity *ω*(*t*), returned by the gyroscope, is corrupted mainly by white and flicker noise. The relative direction change Δ*φ* is calculated by integrating the angular velocity according to Equation (1).

(1) $$\Delta \phi (t)=\int_{0}^{t}\omega (T)dT=\int_{0}^{t}(\hat{\omega }(T)+\omega_{w}(T)+\omega_{f}(T))dT$$

(2) $$\Delta \phi (t)=\Delta \hat{\phi }(t)+\phi_{\text{ARW}}(t)+\phi_{F}(t)$$

(3) $$\phi_{\text{ARW}}(t)=\int_{0}^{t}\omega_{w}(T)dT$$

(4) $$\phi_{F}(t)=\int_{0}^{t}\omega_{f}(T))dT$$

where *ω̂*(*t*) denotes the true angular velocity, *ω_w_* (*t*) white noise and *ω_f_* (*t*) flicker noise in angular velocity readouts. Δ*φ̂*(*t*) is the true change in the heading direction. Integrated white noise *ω_w_*(*t*) results in a first-order random walk, called in this case angular random walk (ARW), and is denoted by *φ*_ARW_(*t*) in Equations (2) and (3). Angular random walk *φ*_ARW_(*t*) is described by the Gaussian distribution but it is not a stationary process as its standard deviation is growing with the square root of the integration time [31]. For the sensor used in the project the standard deviation of *φ*_ARW_ grows at a rate of $2.77{}^{\circ}/\sqrt{\text{h}}$.

Similarly, integrated flicker noise *ω_f_*(*t*) causes an angular error Δ*φ_F_*(*t*), according to Equation (4). The values of Δ*φ_F_* are described by the Gaussian distribution whose standard deviation grows proportionally with the time and therefore it is a non-stationary process. In case of the gyroscope used in the project, the standard deviation of Δ*φ_F_* grows at a rate of 48°/h. Therefore, confidence intervals for estimating Δ*φ*(*t*) widens with the time *t*. After an hour, a motionless gyro can drift by ±144° for ±3*σ* confidence interval. Hence, dead reckoning is justified for short periods only, e.g., during GPS outages.

Inertial sensors also suffer from other errors like non-linearity or temperature random bias. The former error is ±0.3°/s. The experienced random bias due to temperature was much higher than the manufacturer claimed and it reads 0.5°/s for ±3*σ* confidence interval. This is, however, easy to compensate by leaving the sensor motionless and measuring the constant offset to be later subtracted from the readouts.

##### Accelerometer

The accelerometer is used in the project to estimate the user's steps and their lengths. This method is more precise than integrating twice the acceleration readouts to obtain the displacement. The accelerations readouts are also used to estimate the gravity direction which is necessary for correct calculation of the orientation change. By analogy, velocity random walk is a product of integration of white noise in acceleration. On average, the velocity random walk was $0.5\text{m}/\text{s}/\sqrt{\text{h}}$. Flicker noise introduces an error of 9.3 m/s/h. The displacement errors are much larger since they are products of integrating velocity errors. Any offset in acceleration readouts grows quadratically with the time. This quickly leads to aggregation of errors and renders the analytical approach ineffective after a dozen of seconds.

#### 3.1.2. GPS Receiver

The PCB board houses a standard GPS receiver with an integrated ceramic antenna, measuring 15 mm by 15 mm. The receiver is built on the MTK MT3329 chipset and supports WAAS, EGNOS, MSAS, GAGAN corrections. GPS readouts are based on the WGS84 coordinate system aligned in the centre of the Earth, being represented by an ellipsoid. The conversion to a local coordinate system follows in two steps. Firstly, the polar coordinates, *i.e.*, latitude, longitude and height above the ground, are transformed to the WGS84 Cartesian coordinates (*x*_WGS84_, *y*_WGS84_, *z*_WGS84_). This step, along with associated errors and corrections, is described in detail in [32]. Secondly, the local coordinates (*x, y, z*) are obtained by simplified Helmert's transformation (5).

(5) $$\left[\begin{array}{c} x\\ y\\ z \end{array}\right]=\text{R}\left(\left[\begin{array}{c} x_{\text{WGS}84}\\ y_{\text{WGS}84}\\ z_{\text{WGS}84} \end{array}\right]-\text{T}\right)$$

The rotation **R** and translation **T** matrices are structured in such a way that the centre of the local coordinate system is on the Earth's surface. Versor **1**_x_ is directed along with longitude, **1**_y_ with latitude (towards the pole), **1**_z_ is determined by **1**_x_ and **1**_y_. The conversion accuracy is in an order of 10 cm for 20 km from the centre of the local coordinate system.

A GPS receiver provides the HDOP parameter (Horizontal Dilution of Precision) informing about the accuracy of the estimated coordinates, *i.e.*, latitude and longitude (6). Higher values of HDOP should inform a dead reckoning filter to trust to other sources of positioning (e.g., inertial sensors) and disregard GPS readouts.

(6) $$\text{HDOP}=\sqrt{\sigma_{x}^{2}+\sigma_{y}^{2}}$$

The standard deviations of estimating *x* and *y* on the Earth surface are denoted by *σ_x_* and *σ_y_* respectively. Providing that *x* and *y* have the Gaussian distribution and are independent, the probability density function of distance error *r_e_* is given by the Rayleigh distribution [33], which is not a monotone function of its argument. The function has a maximum for *r_e_* > 0. Tests showed that the function defined by Equations (7), (8) and (9) yields definitively superior results. This can be explained by a poor relationship between the HDOP parameter and the error *r_e_*. Since *r_e_* can assume only positive values, the Gaussian function *p*_GPS_(*r_e_, σ*_GPS_) is accordingly scaled by a factor of 2. The coefficient *β*_GPS_ in Equation (9) is chosen by trial and error.

(7) $$p_{\text{GPS}}(r_{e},\sigma_{\text{GPS}})=\frac{2}{\sqrt{2\pi }\sigma_{\text{GPS}}}\exp\left(-\frac{r_{e}^{2}}{2\sigma_{\text{GPS}}^{2}}\right)$$

(8) $$r_{e}=\sqrt{{\tilde{x}}^{2}+{\tilde{y}}^{2}}$$

(9) $$\sigma_{\text{GPS}}=\beta_{\text{GPS}}\text{HDOP}$$

If a GPS receiver loses track of satellites, prediction of its position is based on the previous velocities and position. The first order autocorrelation coefficients for *x̃* and *ỹ* were calculated for a 2.6 km path—see Equation (10). They had considerable values of *a_x_* ≈ *a_y_* ≈ 0.86. Thus *x̃* and *ỹ* can be modelled by Equation (11), which is known as exponentially correlated noise.

(10) $$a=\frac{\overset{n-1}{\underset{t=1}{\text{∑}}}\tilde{x}(t)\tilde{x}(t+1)}{\overset{n-1}{\underset{t=1}{\text{∑}}}\tilde{x}{\left(t\right)}^{2}}$$

(11) $$\left[\begin{array}{c} \tilde{x}(t)\\ \tilde{y}(t) \end{array}\right]=\left[\begin{array}{cc} a_{x} & 0\\ 0 & a_{y} \end{array}\right]\;\left[\begin{array}{c} \tilde{x}(t-1)\\ \tilde{y}(t-1) \end{array}\right]+\left[\begin{array}{c} \upsilon_{x}(t)\\ \upsilon_{y}(t) \end{array}\right]$$

where *υ_x_*(*t*) and *υ_y_*(*t*) are random realizations of white noise, whereby $E\left[\upsilon_{x}^{2}(t)\right]=\left(1-a_{x}^{2}\right)\sigma_{x}^{2}$ and $E\left[\upsilon_{y}^{2}(t)\right]=\left(1-a_{y}^{2}\right)\sigma_{y}^{2}$. This is a very important point. There are periods of e.g., 30s when GPS readouts stray away by 40 m in one direction. A dead reckoning system, weighting GPS coordinates with inertial sensors' readouts to produce a better position estimate, will succumb to this bias error sooner or later. When the GPS receiver regains good visibility with satellites, the estimated position will be all of a sudden in a quite different location. The dead reckoning filter will have to take some time to adapt to a new, accurate GPS position. During this time the filter output will be encumbered with large errors. A way out is to recognize the situation and reinitialize the filter. To account for these random bias errors another source of absolute positioning should be introduced. Maps, video data are examples thereof.

**Figure 3.**
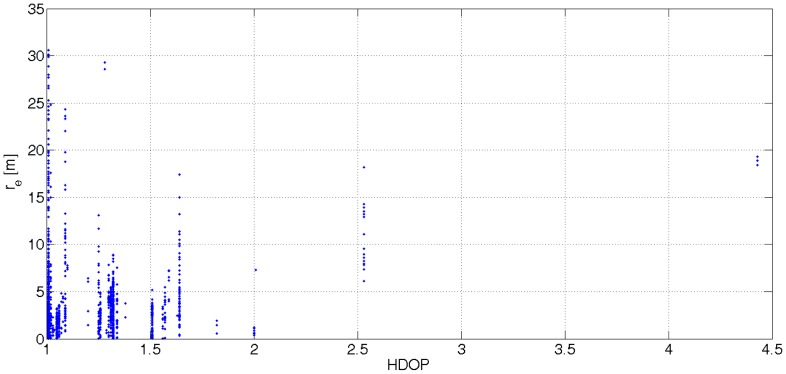
The relation between the values of the HDOP parameter and the distance errors *r_e_*(*t*).

Another problem associated with GPS readouts is the correspondence of the HDOP parameter with the actual accuracy of latitude and longitude. Equation (6) shows that the value of HDOP is interpreted as the distance error. Figure 3 sheds light on the problem. It was noticed that the interpretation of the HDOP parameter depends on a GPS receiver at hand. The correlation coefficient (12) shows a poor linear dependence between distance errors *r_e_*(*t*) and the values of HDOP. The number of observed satellites and position error is not correlated. This confirms the statement about the necessity of additional positioning data.

(12) $$p(r_{e}(t),\text{HDOP}(t))=\frac{\text{Cov}(r_{e}(t),\text{HDOP}(t))}{\sqrt{\text{Var}(r_{e}(t))\text{Var}(\text{HDOP}(t))}}=0.19$$

### 3.2. Pedometer

**Figure 4.**
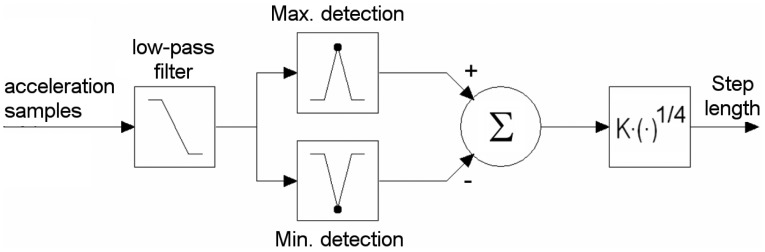
The algorithm for estimating the number of steps and their lengths.

When a person walks, the body undulates in unison with the steps. The step-detection algorithm examines the acceleration of the body only in the vertical direction (parallel to the gravity axis). The gravity axis is obtained from the algorithm presented in Section 3.3. The acceleration signal also reflects knee movements, body swings, sensor jerks and so forth. A 3 Hz 4-order low-pass Butterworth filter was applied to eliminate undesired constituents. The step detection algorithm is presented in Figure 4. The minimum *A*_min_ and maximum *A*_max_ acceleration in the filtered signal are detected. The difference between the peaks is raised to the empirical power of 0.25. The result is multiplied by the scale factor *K*, depending on the individual. Equation (13) expresses an estimated step length d. Related work can be found in [10,13,34]. Step length accuracy measurements are between 1% to 10% depending on an individual and gait style.

(13) $$d={\left(A_{\max}-A_{\min}\right)}^{0.25}\cdot K$$

### 3.3. Tilt Estimation

**Figure 5.**
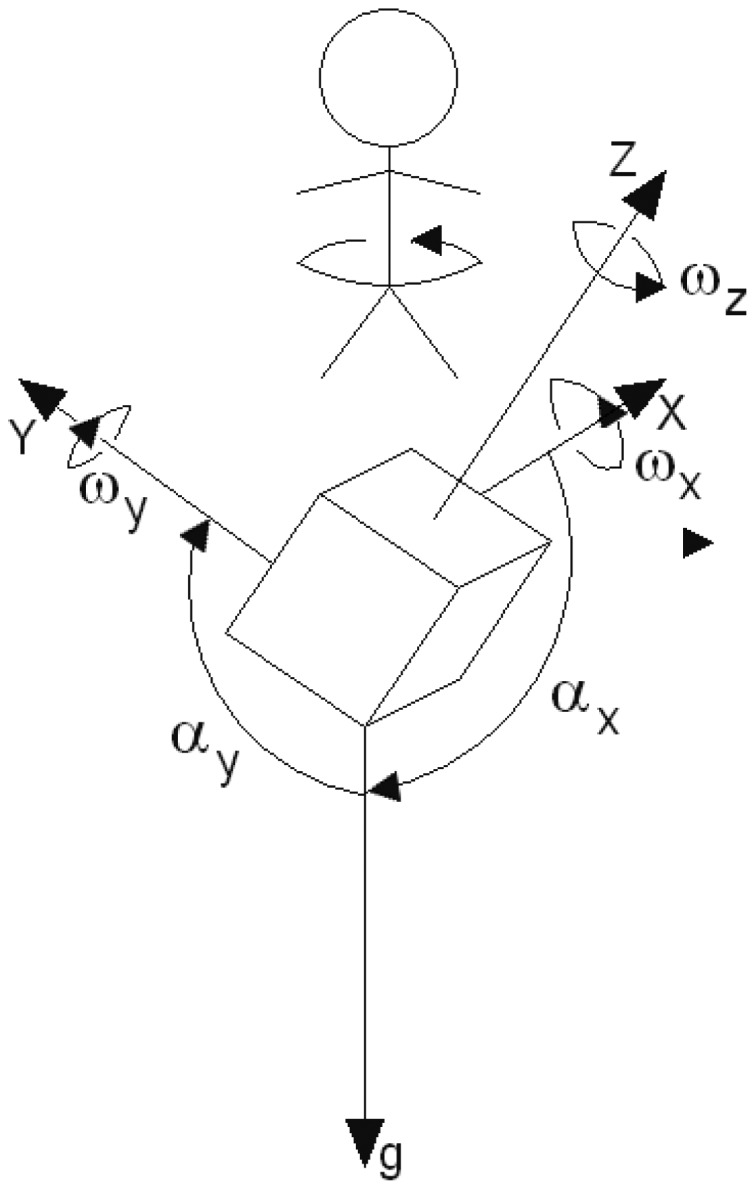
The local coordinate system of the 6DOF sensor. *g* denotes the gravity acceleration. *ω_x_, ω_y_* and *ω_z_* are angular velocities which are clock-wise oriented with their rotation axes. *α_x_, α_y_* and *α_z_* are angles between the sensor's axes and the gravity.

Tilt estimation, *i.e.*, estimation of the direction of the gravity axis, is important for two reasons:

1. Calculation of the relative change in the heading direction. When the sensor is not levelled, the gyroscope's axis does not coincide with the pivotal axis of the pedestrian. When the user turns, the relative change in the heading direction is underestimated. For example, when the gyroscope axis is tilted by 25° from the pivotal axis, the angular velocity is underestimated by ≈ 10%. Consequently, the relative direction change might be 81° instead of 90°—*c.f.* Figure 5.
2. Estimation of the camera orientation with respect to the ground. The stereo camera is used to measure distance to nearest objects like buildings. When the camera is not levelled the measured distance will be overestimated.

A human rotates along the gravity axis when changing the walking direction. Thus, the task of estimating the correct change in direction comes down to finding the gravity axis. The problem is known in aeronautics as Inertial Navigation Systems. The estimation of six coordinates of an aeroplane (three for orientation and three for position) is not a trivial task. A standard approach based on off-the-shelf sensors and cosine direction matrices is very inaccurate after few dozens of seconds [12]. However, humans move with much smaller accelerations. The dominant acceleration comes from the gravity. When a person moves, the body is usually aligned vertically. The pitch and roll are limited to ≈ ±30°. These assumptions help greatly to design an algorithm estimating the gravity axis. The algorithm uses Kalman filter to integrate the readouts from the gyroscopes and accelerometers. The angles between the sensor's axes and the gravity axis (see Figure 5) can be calculated from Equations (14) and (15), given the sensor is not moving.

(14) $$\alpha_{x}=\arccos\left(\frac{a_{x}}{g}\right);\beta_{x}=\frac{\pi }{2}-\alpha_{x}$$

(15) $$\alpha_{y}=\arccos\left(\frac{a_{y}}{g}\right);\beta_{y}=\frac{\pi }{2}-\alpha_{y}$$

(16) $$\alpha_{z}=\arccos\left(\frac{a_{z}}{g}\right)$$

where *a_x_, a_y_* and *a_z_* are accelerations measured by the 3-axial accelerometer, *g* is the gravity constant, 9.81 m/s^2^. When a user walks, the accelerations will fluctuate by ±0.25 g, depending on the walking style. The acceleration vector due to walking is treated here as measurement noise. Hence, calculating the direction of the gravity axis from Equation (16) would result in an error of 15°. The accelerations need to meet the constraint Equation (17).

(17) $$\sqrt{a_{x}^{2}+a_{y}^{2}+a_{z}^{2}}=g$$

This reduces the number of degrees of freedom by one. Gyroscopes can be used to improve the estimation of the sensor orientation. The transition equation of the Extended Kalman Filter (EKF) is given by Equations (18) and (19). Appearance of angular velocity *ω_y_* causes the *X* axis of the sensor changing its orientation with respect to the gravity. This change also depends on the orientation of the *Y* axis and is expressed by the sin *α_y_* term.

(18) $$x(k)=\left[\begin{array}{c} \alpha_{x}(k-1)\\ \alpha_{y}(k-1) \end{array}\right]=\text{f}(\text{x}(k-1),\text{u}(k-1),\text{w}(k-1))=$$

(19) $$=\left[\begin{array}{c} \alpha_{x}(k-1)+\omega_{y}(k-1)+w_{y}(k-1)dT\sin\alpha_{y}(k-1)\\ \alpha_{y}(k-1)-\omega_{x}(k-1)+w_{x}(k-1)dT\sin\alpha_{x}(k-1) \end{array}\right]$$

*k* denotes time instant, where *t* = *k* · *dT* and 1/*dT* = 205 Hz. *w_y_*(*k*) and *w_x_*(*k*) are the gyroscope errors.

The measurement equation of the EKF is given by Equation (20)

(20) $$\text{y}(\text{k})=\left[\begin{array}{c} a_{x}(k)\\ a_{y}(k) \end{array}\right]=\text{g}(\text{x}(k),\text{v}(k))=\left[\begin{array}{c} g\cos\alpha_{x}(k)-\upsilon_{x}(k)\\ g\cos\alpha_{y}(k)-\upsilon_{y}(k) \end{array}\right]$$

**Figure 6.**
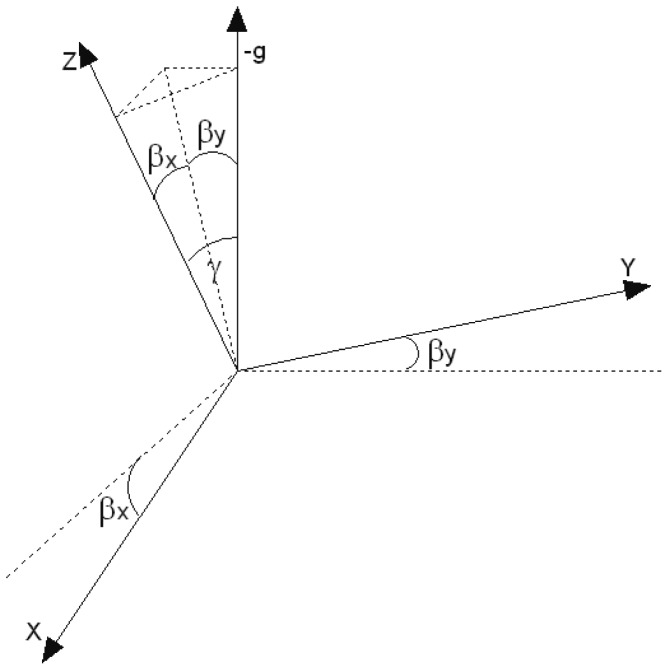
The relationship between the angles *β_x_* and *β_y_* and the angle *γ* which describes the sensor tilt and is measured between the sensor's Z-axis and the −*g* axis.

The accelerometers readouts are corrupted by noise v(*k*). Further implementation of this Extended Kalman Filter is straightforward and follows through Taylor linearisation. Details can be found in [35]. Having estimated *α_x_* and *α_y_* and complementary angles *β_x_* and *β_y_*, the angle *γ* between the *Z*-axis gyroscope and the inverted gravity axis (see Figure 6) can be calculated from Equation (21).

(21) $$\gamma =2\arcsin\frac{\sqrt{\sin^{2}\beta_{x}+\sin^{2}\beta_{y}}}{2}$$

The angular velocity can be corrected by Equation (22). The relative heading direction should be calculated from Equation (23).

(22) $${\hat{\omega }}_{z}(t)=\omega_{z}(t)/\cos(\gamma )$$

(23) $$\Delta \phi (t)=\int_{0}^{t}{\hat{\omega }}_{z}(t)dt$$

**Figure 7.**
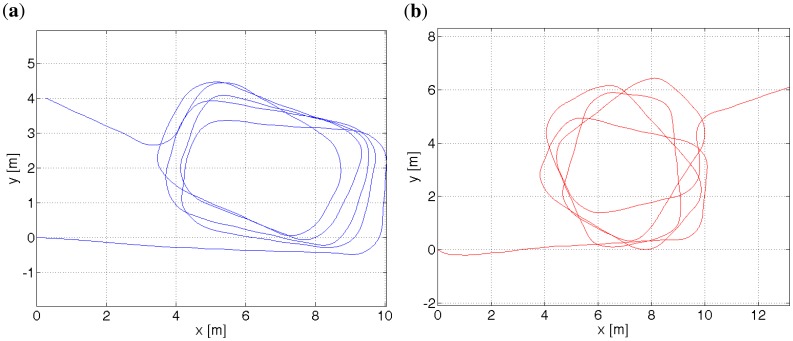
**(a**) Path plotted for the pedometer and gyroscope readouts. The gyroscope readouts are corrected by the gravity estimation algorithm. (**b**) Path with no gyroscope correction. Points (*x, y*) = (0, 0) denote the starting point. There were 20 turns, each 90°. The trial was started and ended in the same point. The difference between the heading direction at the beginning and at the end was ≈ 21°, which corresponds to 1° of error per every rotation.

As mentioned before, this approach is valid for limited range of pitch and roll, due to singularity in Equation (22) when *γ* approaches *π*/2. The measurement of *γ* can be included from Equation (16), however with a small benefit due to considerable fluctuations of the corresponding acceleration *a_z_*. The average error of estimating *γ* was measured to be 0.5° with a standard deviation of 0.45°. Figure 7(a,b) shows a trial with the sensor tilted by ≈15°.

### 3.4. Heading Estimation

A gyroscope provides an angular velocity *ω*. A direction change can be found by integrating *ω* (*c.f.*, Equation (23)). Thus a gyroscope can provide only a relative orientation as opposed to a magnetic compass. Experiments with a magnetic compass showed its susceptibility to magnetic field distortions due to tram lines, DC power lines with no return cable, cars changing a local magnetic field, *etc*. The magnitude of the Earth's magnetic field is *ca.* 50 *μT*. By comparison, a departing tram produces 120 m away a magnetic induction of 5 *μT*. Hence, a magnetic compass was not included. The absolute pedestrian orientation is sorted out run-time by the particle filtering algorithm based on other data. A magnetic compass could give a rough approximation ±90° until the absolute orientation has been found and then switched off.

### 3.5. Probability Map

The digital map is divided into three types of area. The areas have their associated weights:

- forbidden areas—buildings, ponds, walls, fences *etc.*, *w*_map_(*x, y*) = 0.0,
- probable areas—lawns, fields *etc.*, *w*_map_(*x, y*) ∈ (0; 1),
- preferred areas—pavements, streets, squares, alleys *etc.*, *w*_map_(*x, y*) = 1.0.

**Figure 8.**
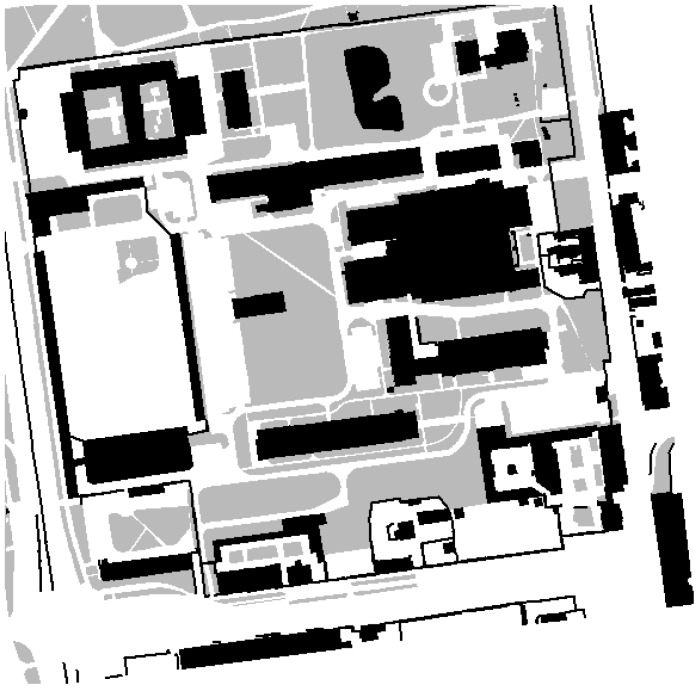
An example of a probability map of the University Campus. Forbidden, probable and preferred areas are denoted by black, grey and white respectively. The streets at the campus are paved and have the same texture as pavements. A blind user would rather refrain from walking through lawns.

*w*_map_(*x, y*) denotes the type of an area at (*x, y*) coordinates. Each weight corresponds to the probability of the user being in a given area. Since the system is destined for outdoor navigation, it is rather improbable that the user will traverse buildings, walls and fences—*c.f.*, Figure 8. The user is likely to travel along pavements and alleys, however, he or she can also walk across lawns, although less likely. The probability map mitigates the problem of gyroscope drifts and stepmeter inaccuracy. The particle filtering approach to the discussed problem will be explained in Section 3.9. Here the term particles denotes a collection of hypothetical locations of the user and the weights associated with each particle is understood as the probability of the user being at this location. When particles enter a forbidden area, they will be gradually eliminated. Particles that move along a preferred area will be preserved. Therefore, the direction will align itself and the direction drift will be reduced. The weight assigned for probable areas should not be too low, because otherwise when a user crosses a wide street, the particles' weights will be successively reduced and consequently die out. Consequently, the user's location will not be correctly calculated. The project was developed with a view of pedestrians, especially blind ones.

### 3.6. Stereovision Camera

#### 3.6.1. Introduction

**Figure 9.**
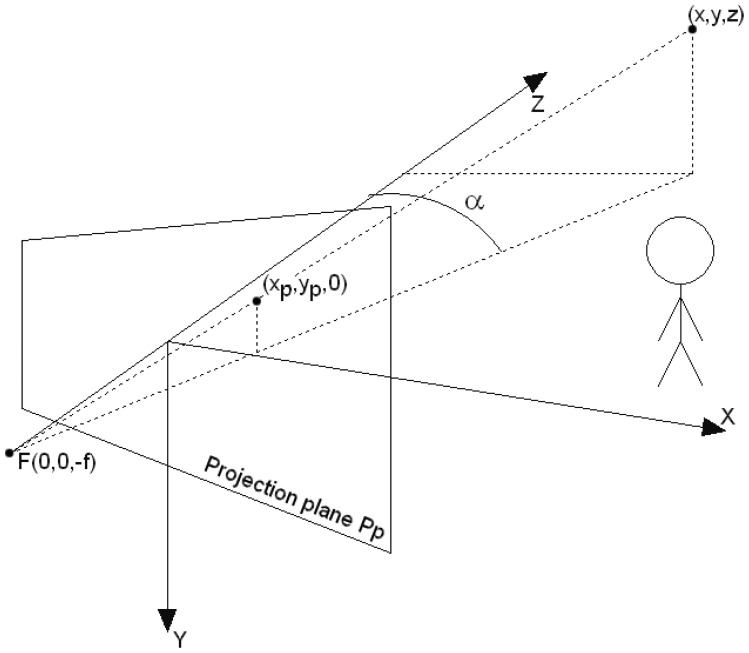
The local coordinate system of the stereovision camera. *F* is the focus of the camera, and f is the focal length, (*x,y,z*) are the coordinates of a point in the camera coordinate system. This point has the coordinates (*x_P_, y_P_*, 0) on the projection plane *P_P_* of the camera and *α* denotes the angle from the *Z* axis at which point (*x, y, z*) is visible by the camera.

A stereovision camera [28] is comprised of two cameras which are shifted away along the *X* axis, *c.f.*, Figure 9. The distance between the cameras is called the baseline of a stereo system and is denoted by *B*. The baseline distance makes the same scene point *P* to be visible in the left and right camera at coordinates *x_L_* and *x_R_* respectively. The difference between the *x_L_* and *x_R_* values is called the disparity, and will be denoted by *x_d_*. The z coordinate of the observed object can be calculated from Equation (24), where *f* is the focal length.

(24) $$z=\frac{Bf}{x_{d}}$$

For the legibility sake, the stereovision system can be treated as one camera, whose simplified coordinate system is shown in Figure 9. The *Z* axis coincides with the optical axis of the camera model.

The coordinates of a point (*x, y, z*) are projected onto the camera projection plane *P_P_* according to the transformation (25).

(25) $$\left[\begin{array}{c} x_{p}\\ y_{p} \end{array}\right]=\left[\begin{array}{ccc} \frac{f}{z+f} & 0 & 0\\ 0 & \frac{f}{z+f} & 0 \end{array}\right]\;\left[\begin{array}{c} x\\ y\\ z \end{array}\right]$$

The depth of a point (*x, y, z*), denoted by *d_p_*(*x, y, z*), is understood as the distance from the point to the projection plane and equals Equation (26).

(26) $$d_{p}(x,y,z)=z$$

The distance from the Y axis of the camera to a point with the (*x, y, z*) coordinates is denoted by *d_Y_* (*x, y, z*) and calculated from Equation (27).

(27) $$d_{Y}(x,y,z)=\sqrt{x^{2}+z^{2}}$$

Let us define a function which returns the distance from the *Y* camera's axis to the nearest point visible at angle *α* from the optical axis *Z, c.f.* Figure 9. This function is defined by Equation (28).

(28) $$d_{\alpha }(\alpha )=\min(d_{Y}(x,y,z));\text{where}\tan\alpha \frac{x_{p}}{f}=\frac{x}{z}$$

The data from the stereovision camera is digitized. The (*x_p_, y_p_*) coordinates are quantized and converted to units called pixels. The transformed coordinates are denoted by (*x*_pix_, *y*_pix_). The conversion follows through Equation (29).

(29) $$\left[\begin{array}{c} x_{\text{pix}}\\ y_{\text{pix}} \end{array}\right]=\left[\begin{array}{c} \text{Round}\left(\frac{f_{\text{pix}}}{f}x_{p}\right)\\ \text{Round}\left(\frac{f_{\text{pix}}}{f}y_{p}\right) \end{array}\right]$$

where the Round() operator denotes rounding to a nearest integer number, *f*_pix_ is the focal length of the camera expressed in pixels and is provided by the manufacturer in the technical specification. The difference between the maximal and minimal value of *x*_pix_ is called the horizontal resolution of the camera and is denoted by *x*_res_. The vertical resolution *y*_res_ is defined by analogy. A depth map or a depth picture **L** is a two dimensional map defined by Equation (30).

(30) $$\text{L}(x_{\text{pix}},y_{\text{pix}})=\min(d_{p}(x,y,z));(x,y)\overset{\left(25),(29\right)}{\to }(x_{\text{pix}},y_{\text{pix}})$$

The coordinates (*x, y*) are related with (*x*_pix_, *y*_pix_) through transformations (25) and (29). Equation (30) formally says that a stereo camera provides the depth to nearest points that are not occluded. This is so-called 2.5 dimension image. Usually, the depth for a given point (*x*_pix_, *y*_pix_) is represented by a floating point-number. Using the identity (24), the disparity map **K** can be defined by Equation (31).

(31) $$\text{K}(x_{\text{pix}},y_{\text{pix}})=\frac{Bf}{\text{L}(x_{\text{pix}},y_{\text{pix}})}$$

As a matter of fact, stereovision cameras first determine the disparity map **K** and then calculate the depth map **L**. The disparity *x_d_* is calculated in digital cameras in pixels, thus **K** should be a map containing integer numbers. An example of a depth map is presented in Figure 12(b). Advanced stereo cameras perform subpixel interpolation, e.g., the camera used in the project calculates the disparity with the resolution of 0.25 pixel and then performs filtering and interpolation on the depth map.

#### 3.6.2. Error Analysis

**Figure 10.**
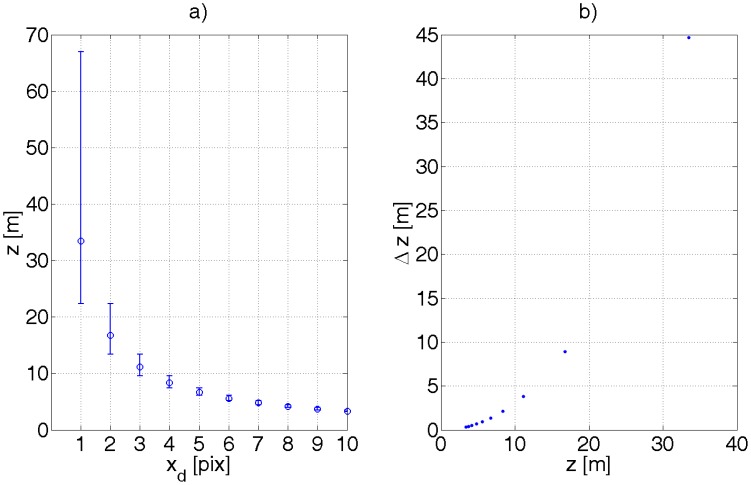
An illustration of the quantization effect on estimating the *z* coordinate. (**a**) The plot of *z* and errors thereof as a function of disparity *x_d_*. Dots symbolize the depth *z* calculated from *z* = *Bf/x_d_*. Bars symbolizes errors of *z* when *x_d_* is encumbered with an error of $\pm \frac{\in }{2}$ or $\pm \frac{\text{pixel}}{2}$, (**b**) The absolute error of *z* as a function of the depth *z*.

As the object recedes from the camera, the disparity *x_d_* decreases. An error in calculating the shift *x_d_* becomes more significant. Calculating the derivative of *z* with respect to *x_d_* in Equation (24) gives Equation (32). The accuracy of determining the distance *z* decreases with its squared value. As the stereo-camera is digital, the values of *x_L_, x_R_* are quantized and so is *x_d_*. It means that *z* manifests discontinuities as *x_d_* changes to an adjacent quantum. Let ∈ denote the size of the pixel in metrical units. For the sake of simplicity, *x_d_* will have units of metres or pixels, whereby the appropriate unit will be clear from the context or mentioned implicitly. Let us assume at this stage that the error of determining x*_d_* can be $\pm \frac{\in }{2}$ or $\pm \frac{\text{pixel}}{2}$. Figure 10 exemplifies the effect of such quantization.

(32) $$\Delta_{z}\approx -\frac{Bf}{x_{d}^{2}}\Delta x_{d}\approx -\frac{z^{2}}{Bf}\Delta x_{d}$$

The error of determining the disparity does not only come from quantizing x_d_, in which case, the disparity maps presented in Section 3.8 would be devoid of any artifacts or falsely calculated disparities—see [36] for full clarification.

The camera used in this work calculates disparity by correlating small blocks (e.g., 7 × 7 pixels) and looking for best match. The probability density function of the disparity error is disputable in this case. Let it be the Gaussian distribution, expressed by Equation (33), where Δ*x_d_* denotes the disparity error.

(33) $$f_{d}(\Delta x_{d})=\frac{1}{\sqrt{2\pi }\sigma_{d}}\exp\left(-\frac{\Delta x_{d}^{2}}{2\sigma_{d}^{2}}\right)$$

The probability density function of Δ*z* can be calculated from Equation (34). The function *f_z_* is conditioned on *z, i.e.*, the error Δ*z* depends on the value of *z* as mentioned before.

(34) $$f_{z}(\Delta z|z)=\left|\frac{\partial (\Delta x_{d})}{\partial (\Delta z)}\right|f_{d}(x_{d})$$

Equation (32) holds for small *x_d_*. The exact value of Δ*z* can be computed from Equation (35). The formula necessary for the transformation is given by Equation (36).

(35) $$\Delta z=\frac{Bf}{x_{d}+\Delta x_{d}}-z$$

(36) $$\frac{\partial (\Delta x_{d})}{\partial (\Delta z)}=\frac{Bf}{{\left(\Delta z+z\right)}^{2}}$$

Thus, *f_z_* can be calculated from Equation (37).

(37) $$f_{z}(\Delta z|z)=\frac{Bf}{{\left(\Delta z+z\right)}^{2}}\frac{1}{\sqrt{2\pi }\sigma_{d}}\exp\left(-\frac{{\left(Bf\Delta z\right)}^{2}}{2\sigma_{d}^{2}{\left(z+\Delta z\right)}^{2}}\right)$$

**Figure 11.**
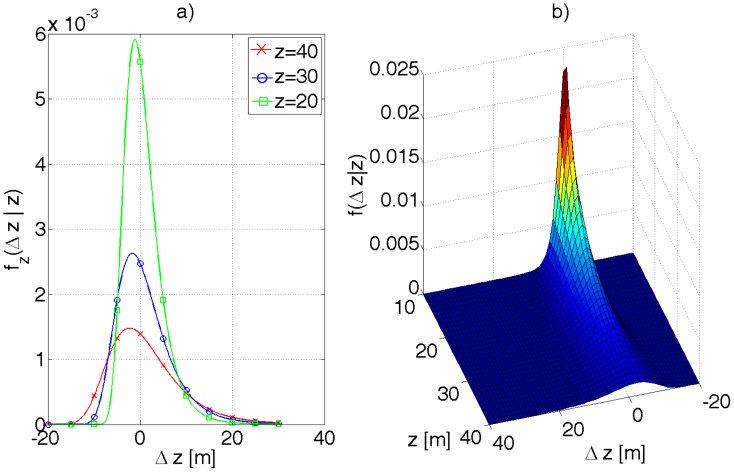
(**a**) A plot of *f_z_*(Δ*z*|*z*) for different values of *z*. As *z* increases the conditional probability density function widens. (**b**) A 3D plot of *f_z_*. For illustration purposes, *σ_d_* was assumed 6 pix, whereby, to account for the units, *f* = 279 pix was substituted.

The shape of the function *f_z_*(Δ*z*\*z*) for different values of *z* is presented in Figure 11. The function is skewed as one should have suspected and decays slower for Δ*z* greater than 0.

### 3.7. 3D Map of an Urban Terrain

A precise 3D map of an urban terrain was provided by the local cartographic office of Lodz (MODGiK). The map is divided in layers containing: roads, buildings, entrances to buildings, steps, fences, lawns, trees, bushes, ponds, lamp posts and many other data not used in the project. The accuracy of building deployment is in an order of single centimetres and the buildings' heights were measured with a bit worse accuracy. The radius of trees' crowns was assumed 3 m and the radius of lamp posts 0.5 m.

### 3.8. 3D Matching Algorithm

Estimated location based on the stepmeter, gyroscope and digital maps is accurate to within 15 m (a street with pavements on both sides) [3]. GPS errors in urban canyons being a several dozens of metres, practically both sides of a road are equally probable.

**Figure 12.**
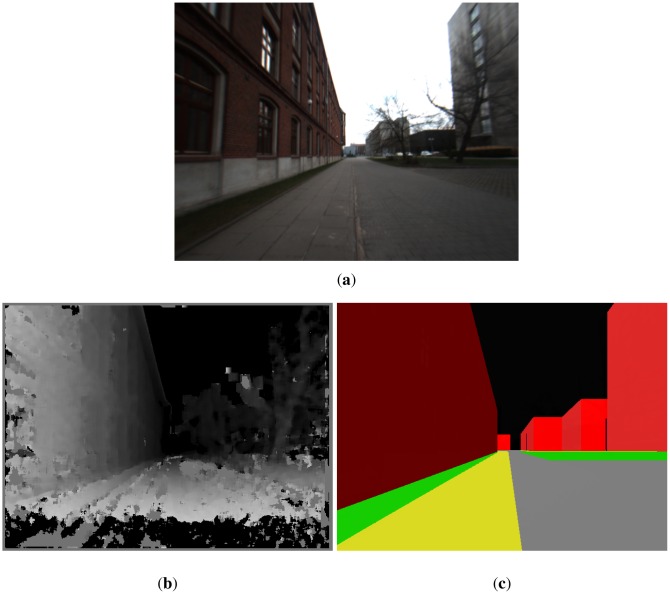
(**a**) TV camera image. (**b**) Disparity map from the stereo camera. Darker colours correspond to smaller disparity, thus bigger distance. (**c**) 3D model of the corresponding urban environment.

Figures 12(a–c) show a picture from a monocamera, a disparity map **K** and a part of the 3D model of the University Campus.

Let us use the local coordinate system defined in Section 3.1.2. Let the vector [*x*′*_S_, y*′*_S_, z*′*_S_, φ*′*_S_*]*^T^* denote the Cartesian coordinates and the orientation of the stereo camera in the local coordinate system. *φ*′*_S_* is called the azimuth of the stereo camera and it has the same interpretation as with a magnetic compass. It is assumed that the stereovision camera is worn by a pedestrian and it is attached to the chest, say at *z* = *h* = 1.5 m above the ground. The optical axis of the camera is directed roughly parallel to the ground plane. The positioning algorithm has a precise 3D model of the environment that is defined for the local coordinate system—see Figure 12(c). Let us put a virtual camera in the (*x*′*_V_, y*′*_V_, z*′*_V_*) coordinates and set its orientation to *φ*′*_V_*. Then, let us render the virtual environment based on the 3D model. The virtual camera should see the picture presented in Figure 12(c). On the other hand, the stereovision camera provides a depth image of the environment. Both images can be compared. Let **L_S_** and **L_V_** denote the depth image returned by the stereovision camera and retrieved from the virtual environment, correspondingly. *f_L_*(**L_S_, L_V_**) is some cost function comparing the two depth images and returning comparison error. The function is defined by Equation (38).

(38) $$f_{\text{L}}:(\text{L},L)\to {\mathbb{R}}^{1}$$

Formally, the problem comes down to finding the vector [*x*′*_V_, y*′*_V_, z*′*_V_, φ*′*_V_*]*^T^* where the function *f_l_*(**L_S_, L_V_**) returns a minimum value, that is, the difference ‖**L_S_** − **L_V_**‖ is minimal according to some criterion. Then the estimated coordinates of the stereo camera (that is the pedestrian location) are given by Equation (39). This task is handled by the particle filtering algorithm.

(39) $${\left[x_{S}^{\prime },y_{S}^{\prime },z_{S}^{\prime },\phi S\right]}^{T}={\left[x_{V}^{\prime },y_{V}^{\prime },z_{V}^{\prime },\phi_{V}^{\prime }\right]}^{T}$$

**Figure 13.**
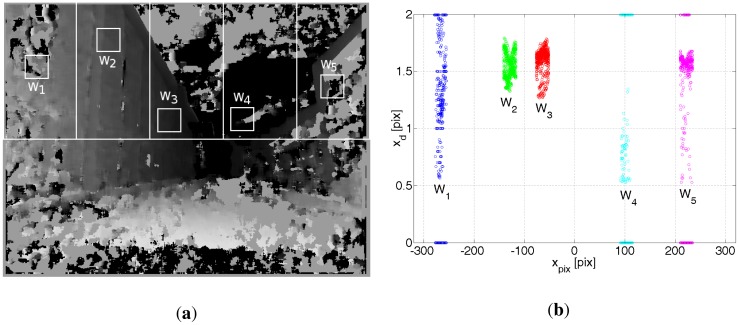
(**a**) An example of a disparity map **K**. Darker colours correspond to smaller values of disparity, thus larger distances. Example windows for each region *S_j_* (*j* = 1… 5) are denoted by **W**_1_ through **W**_5_. One can see that for windows **W**_2_ and **W**_3_ the depth map was determined correctly and these windows should be compared with corresponding windows in the **L***_V_* depth map. (**b**) The disparity values *x_d_* for each window **W**_j_ as a function of the *x*_pix_ coordinate in the disparity picture. For **W**_1_, **W**_4_ and **W**_5_ windows, the disparity *x_d_* changes from 0 pix to ≈2 pix which corresponds to distances ∞ to ≈16 m. These windows should be rejected from further analysis.

Assuming *z_S_* = *z_V_* = *h* reduces the dimension of the problem. The function *f_L_* is still not trivial. The depth picture **L_S_** is encumbered with many errors, see Figure 12(b). For example, sun illumination causes some parts of the depth map to have falsely determined values (see Figure 13). The function *f_L_* should be immune to these undesirable effects that can cause random bias errors, most troublesome errors to correct by any filter, including a particle filter which is used in the presented project. A random bias error is understood as an error whose expected value differs considerably from 0 for a significant period of time, e.g., 30 seconds. There can be also objects that can compromise the comparison between **L_S_** and **L_V_** and cannot be filtered by any methods, *i.e.*, parked cars, trucks, bushes, posters, garbage bins, *etc*. Their influence can be minimized to some extent by excluding from comparison that part of **L_S_** which is below the optical axis of the camera. Hence the ground surface is also excluded from comparison. First **L_S_** is analysed to seek fragments for comparison. The method of analysing **L_S_** depends on many empirical factors that were verified during many trials. The idea is following:

1. Reject the part of the depth map **L_S_** that is below the optical axis of the camera.
2. Divide vertically the upper part of **L_S_** into *n_S_* = 5 equal regions *S_j_, j* = 1… *n_S_*—see Figure 13(a). This forces to compare the depth maps *L_S_* and *L_V_* in different regions, which, to some extend, decorrelate random bias errors.
3. for *j* = 1 to *n_S_*
  - From the area *S_j_*, pick at random a small square window **W_j_** of *W_W_* by *W_W_* measurements, where *W_W_* = 24 pix.
  - Check if **W***_j_* is devoid of artifacts like sun illumination, falsely determined disparity *etc.*—*c.f.* Figure 13(b).
  - Calculate the angle *α_j_* at which the camera sees the object pointed by **W***_j_* (compare with the angle *α* in Figure 9).
  - Calculate the distance *d_Sj_* to an object encompassed by **W***_j_*.
  - Check what the virtual camera sees at the angle *α_j_* from coordinates [*x*′*_V_, y*′*_V_, z*′*_V_, φ*′*_V_*]. Calculate in this direction the distance to the nearest object *d_Vj_*.
  - Compare the distances *d_Sj_* and *d_Vj_* by the distance error function *f_Lj_*.
4. Return the comparison error between **L_S_** and **L_V_** as *f_L_*(**L_S_, L_V_**) = *f_L_*_1_ · *f_L_*_2_ · *f_L_*_3_ · *f_L_*_4_ · f*_L_*_5_.

**Ad. 1, 2.** The areas S*_j_* are shown in Figure 13(a).

**Ad. 3a.** The window **W***_j_* is picked at random from the corresponding area S*_j_*, according to Figure 13(a).

**Ad. 3b.** By trial and error, the empirical measure (40) was proposed to check if the disparity map for **W***_j_* is devoid of artifacts. $x_{d}^{\max}$ (41) and $x_{d}^{\min}$ (42) are the maximum and minimum values of disparities for a given window **W***_j_*

(40) $$k_{j}=\frac{x_{d}^{\max}({\text{W}}_{j})-x_{d}^{\min}({\text{W}}_{j})}{x_{d}^{\max}({\text{W}}_{j})}$$

(41) $$x_{d}^{\max}({\text{W}}_{j})=\max(\text{K}(x_{\text{pix}},y_{\text{pix}}));\forall (x_{\text{pix}},y_{\text{pix}})\in {\text{W}}_{j}$$

(42) $$x_{d}^{\min}({\text{W}}_{j})=\min(\text{K}(x_{\text{pix}},y_{\text{pix}}));\forall (x_{\text{pix}},y_{\text{pix}})\in {\text{W}}_{j}$$

Window **W***_j_* is stated to be devoid of artifacts if *k_j_* > *k*_thr_, where an empirical value of *k*_thr_ ≈ 0.8.

**Ad. 3c.** The angle *α_j_* at which the stereovision camera sees the object encompassed by the **W***_j_* window can be calculated from Equation (43), where *x*^(c)^ denotes the *x* coordinate of the centre of **W***_j_*.

(43) $$\alpha_{j}=\arctan\frac{x_{j}^{\left(c\right)}}{f_{\text{pix}}}$$

**Ad. 3d.** The distance *d_Sj_* to the object pointed by **W***_j_* can be calculated from Equation (44). This is an arithmetical average of depths divided by the cos *α_j_* factor to obtain the Euclidean distance—*c.f.*, Equations (26) and (27) that show the difference between the depth of a point and distance to the camera's *Y* axis.

(44) $$d_{{\text{S}}_{j}}(\alpha_{j})=\frac{1}{\cos\alpha_{j}}\frac{1}{W_{W}^{2}}\sum_{x_{\text{pix}}}\sum_{y_{\text{pix}}}{\text{L}}_{S}(x_{\text{pix}},y_{\text{pix}});(x_{\text{pix}},y_{\text{pix}})\in {\text{W}}_{j}$$

**Ad. 3e.** Given the coordinates and orientation of a virtual camera in the 3D environment, the *d_Vj_* distance from the virtual camera to the nearest object at *α_j_* angle can be easily obtained.

**Figure 14.**
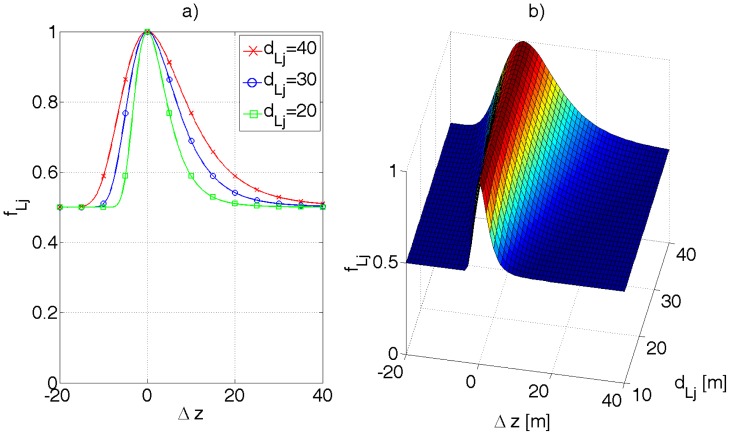
(**a**) An example of the *f_Lj_* function from Equation (45) where *k_j_* > *k*_thr_. Example values of *a* = 0.5 and *σ_L_* = 6 pix were used (to account for the units, *f* = 279pix was substituted). (**b**) A 3D plot of *f_Lj_* function where *k_j_* > *k*_thr_ and Δ*z* = *d_Sj_* − *d_Lj_*. The base of the function widens as the compared distances increase.

**Ad. 3f.** The corresponding distances *d_Sj_* and *d_Vj_* are compared by the error function *f_Lj_*. For the integrity sake of Equation (47), the function *f_Lj_* should return 1 if window **W***_j_* was rejected from comparison, *i.e., k_j_* ≤ *k*_thr_. The function *f_Lj_* reflects the correspondence between *d_Sj_* and *d_Vj_*, whereby the comparison should be more tolerant for greater distances, according to formula (32)—the accuracy of determining *d_Sj_* drops with its square value. Following the reasoning in the previous chapter, the function *f_Lj_*, expressed by Equation (45), was proposed. The parameter *a* introduces some tolerance in case of unexpected objects registered by the stereo camera (e.g., cars, humans), *c.f.* Figure 14. Experimentally, *a* ∈ < 0.95, 0.98 > gives desired results.

(45) $$f_{Lj}(k_{j},d_{S_{j}},d_{V_{j}})=\left\{\begin{array}{ll} 1 & k_{j}\leq k_{\text{thr}}\\ a+(1-a)\exp\left(-\frac{{\left(Bf\Delta z\right)}^{2}}{2\sigma_{L}^{2}{\left(d_{L_{j}}+\Delta z\right)}^{2}}\right) & k_{j}>k_{\text{thr}} \end{array}\right\}$$

(46) $$\Delta z=d_{S_{j}}-d_{L_{j}}$$

**Ad. 4.** Finally, the function *f_L_* comparing the two environments, *i.e.*, real and virtual, can be defined by Equation (47).

(47) $$f_{\text{L}}({\text{L}}_{\text{S}},L_{\text{V}})=\prod_{j=1}^{n_{s}}f_{L_{j}}(k_{j},d_{S_{j}},d_{V_{j}})$$

The 3D match algorithm is evoked 10 times a second.

### 3.9. Particle Filtering

#### 3.9.1. Introduction

The particle filter is a sequential version of the Monte Carlo method [37] which enables to find solutions of a multidimensional problem by generating a large number of possible solutions, so-called particles, and then verifying these solutions by a given criterion, *i.e.*, an error function. The particle filter can be looked at from the statistical point of view, whereby the solution is represented by a probability density function. The particle filter improves upon Kalman filter in case of multi-modal distributions and non-linear dynamics at the cost of high computation burden. Let *i* denote the number of a particle and *L* the total number of particles, thus *i* ∈ < 1, *L* >. A particle at a given time instant *k* is represented by vector c*_i_*(*k*) given by Equation (48).

(48) $${\text{c}}_{i}(k)=\left[\begin{array}{c} {\text{x}}_{i}(k)\\ w_{i}(k) \end{array}\right]$$

where x*_i_*(*k*) is a possible system state at time instant *k* and *w_i_*(*k*) is the weight associated with *i*-th particle. A possible system state is understood as a possible solution of the problem in question. In our case x*_i_*(*k*) represents a hypothetical user location and orientation, x*_i_*(*k*) = [*x_i_, y_i_, φ_i_*]*^T^, x_i_, y_i_* denote coordinates in the local coordinate system defined in Section 3.1.2. *φ_i_* is the azimuth orientation. The aim of this simulation is to to find the most probable user location. Given the series of measurements y(1) … y(*k*) the pedestrian's location is given by the probability density function that can be approximated by the probability mass function (49).

(49) $$p(\text{x}(k)|\text{y}(1:k))\approx \sum_{i=1}^{L}\delta (\text{x}-{\text{x}}_{\text{i}}(k))\cdot w_{i}(k)$$

where *δ*(·) is the Dirac delta function. Weights *w_i_*(*k*) of all generated particles should sum up to unity.

#### 3.9.2. Implementation of the Particle Filtering Algorithm

The implementation of the particle filtering algorithm for processing the positioning data is as follows:

1. **Initialization.** At the algorithm outset for *k*=1, all the particle states x_i_(1) are randomly initialized according to a given distribution. The weights *w_i_*(1) are assigned equal values of $\frac{1}{L}$.
2. **Prediction.** Based on the transition Equation (50), new particle states are predicted.
  (50) $${\text{x}}_{i}(k)=\text{f}({\text{x}}_{i}(k-1),\text{u}(k-1),{\text{v}}_{i}(k-1))$$
  u(*k* − 1) is a driving vector, v*_i_*(*k* − 1) is a noise vector introduced to the state due to the error of u(*k* − 1), where each particle is perturbed with an individually generated vector v*_i_*(*k* − 1), f(·) is a transition function that calculates a new state based on the previous one, driving signals and errors thereof.
3. **Measurement update.** Each measurement y(*k*) updates the weights of the particles by Equation (51).
  (51) $$w_{i}(k)=w_{i}(k-1)\cdot p(\text{y}(k)|{\text{x}}_{i}(k))$$
  *p*(y(*k*)|x*_i_*(*k*) is a conditional probability density of measuring y(*k*), given the particle state x*_i_*(*k*). In other words, *p*(y(*k*)|x*_i_*(*k*) characterizes error distribution of a given data source. Particles that diverge in the long run from measurements will assume small weights *w_i_*(*k*).
4. **Weights' normalization.** The weights are normalized so that they sum up to 1—Equation (52).
  (52) $$w_{i}(k):=\frac{w_{i}(k)}{\overset{L}{\underset{i=1}{\text{∑}}}w_{i}(k)}$$
5. **State estimation.** The most probable system state is estimated, e.g., by the first moment (53):
  (53) $$\bar{\text{x}}(k)=\sum_{i=1}^{L}{\text{x}}_{i}(k)\cdot w_{i}(k)$$
6. **Resampling.** After a number of algorithm iterations, all but a few particles have negligible weights and therefore do not participate in the simulation effectively. This situation is detected by calculating the so-called degeneration indicator, expressed by Equation (54), which is an inverse of the second moment reflecting the dispersion of the weights.
  (54) $$d(k)=\frac{1}{L\overset{L}{\underset{i=1}{\text{∑}}}w_{i}^{2}(k)}$$
  If all particles have the same weights of *L*^−1^ then *d*(*k*) reaches its maximum value of 1. As the weights start to differ, the *d*(*k*) indicator decreases. If *d*(*k*) falls below a given threshold, then a process called resampling is introduced and a new set of particles is created. The probability of copying a particle to the new set is proportional to its weight *w_i_*(*k*). Therefore particles that poorly approximate the system state are superseded by more “accurate” particles. Then all particles are assigned the same weight *L*^−1^.
7. Go to point 2.

#### 3.9.3. Implementation

**Ad. 1.** The particles can be initialized, e.g., around a first GPS readout.

**Figure 15.**
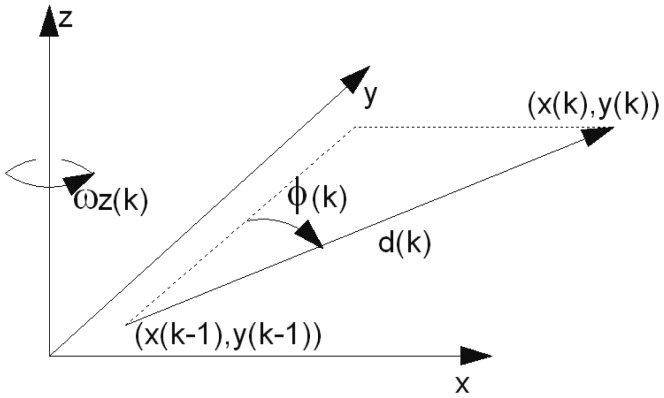
An explanation of the unperturbed state transition: *d*(*k*) is the length of a step estimated by the pedometer. *φ*(*k*) is the azimuth orientation. *ω_z_*(*k*) is the angular velocity around the gravity axis. *ω_z_* is estimated by the gyroscope and the gravity estimation algorithm. (*x*(*k* − 1),*y*(*k* − 1)), (*x*(*k*), *y*(*k*)) are the coordinates in the previous and current time instant.

**Ad. 2.** Based on Figure 15, the transition equation for a particle *i* reads (55).

(55) $$\left[\begin{array}{c} x_{i}(k)\\ y_{i}(k)\\ \phi_{i}(k) \end{array}\right]=\left[\begin{array}{c} x_{i}(k-1)\\ y_{i}(k-1)\\ \phi_{i}(k-1) \end{array}\right]+\left[\begin{array}{c} \left(d(k)+\upsilon_{i}(k))\sin(\phi_{i}(k-1)-(\omega_{z}(k)+\omega_{i}(k))dT\right)\\ \left(d(k)+\upsilon_{i}(k))\cos(\phi_{i}(k-1)-(\omega_{z}(k)+\omega_{i}(k))dT\right)\\ -(\omega_{z}(k)+\omega_{i}(k))dT \end{array}\right]$$

*υ_i_*(*k*) and *w_i_*(*k*) are random realizations of the pedometer and gyroscope noise respectively. Each particle is perturbed individually. *k* is the time instant, where *t* = *k* · *dT*.

**Ad. 3.** The following sources are used to update the particle weights:

- GPS. The measurement update for the GPS readouts are given by Equations (56) through (58) based on the Equation (7)
  (56) $$w_{i}(k)=w_{i}(k-1)\cdot p_{\text{GPS}}(r_{i},\sigma_{\text{GPS}})$$
  (57) $$r_{i}=\sqrt{\left({\left(x_{\text{GPS}}(k)-x_{i}(k)\right)}^{2}+{\left(y_{\text{GPS}}(k)-y_{i}(k)\right)}^{2}\right)}$$
  (58) $$\sigma_{\text{GPS}}=\beta_{\text{GPS}}\text{HDOP}(58)$$
  *β*_GPS_ is a coefficient which enables to weight GPS readouts with appropriate importance. Larger values of *β*_GPS_ makes the *p*_GPS_() function more tolerant to GPS errors.
  (59) $$w_{i}(k)=w_{i}(k-1)\cdot w_{\text{map}}(x_{i}(k),y_{i}(k))$$
- comparison between the 3D virtual environment model and depth picture retrieved from the stereo camera. The measurement update equation is given by Equation (60).
  (60) $$w_{i}(k)=w_{i}(k-1)\cdot f_{L,i}(L_{S},L_{V,i})$$
  For each particle, a virtual environment is generated individually. The coordinates of the virtual camera are set to [*x_i_, y_i_, h, φ_i_*]. *h* is the height at which the stereo camera is attached. Then the depth map *L_V,i_* is generated. The function *f_L_*(**L_S_, L_V,i_**) is evoked for every particle. This is a computation demanding procedure. Note that, if an unexpected object appears in front of the stereo camera for 10 seconds, given particles' weights will be decreased by *a*^10/^*^s^*^·10^*^s^, c.f.* Equation (45) (3D matching algorithm is run 10 times a second). This might introduce a large error in the position estimation. Bigger values of *a* diminish the benefit of using a stereo camera and 3D model of the urban terrain.

## 4. Trials

The trials were carried out at the campus of Lodz University of Technology, in an area of *ca.* 500 m by 500 m. The length of the trial path was *ca.* 2.6 km. The reference path was restored off-line by analysing images recorded by the camera. The average error of determining the reference path was *ca.* 0.5 m on average. The maximum error might have been 2–3 m. The delineated path is composed of straight lines. The location error is calculated as the distance between the appropriate path segment and the estimated location by a GPS receiver or the particle filter. Thus, actual positioning errors are larger. On the other hand, GPS errors assume dominant values in the direction perpendicular to a street. In the direction parallel to a street there are no buildings to occlude the sky. Hence, the signal from GPS satellites is not occluded, which results in a small error in the direction along a street [2].

## 5. Results

The results are presented by plots of paths, a histogram and a cumulated histogram of errors and a table that summarizes the errors. An interesting question is how much a given data source improves the accuracy of the positioning. Table 1 provides the answer. Therefore, several off-line tests were run with different combination of data sources. It was though assumed that the starting location is known. Otherwise, a bigger number of particles would have been necessary to find a first approximate location. Once, a rough location is found the filter is convergent. The initial errors might have been large and therefore eclipse the key point of measurements. On one side, this assumption might be justified. A pedestrian, e.g., a blind user goes out from his home, so he knows his location. On the other hand, a blind pedestrian might use the system when lost. The aforementioned distance error *r_e_* is used as a comparison criterion. The results are evaluated by the following measures:

1. Root-mean-square value, denoted by RMS*_e_*,
2. Mean error, denoted by *μ_e_*,
3. CEP50—Circle of Error Probability 50%—the radius of a circle encompassing 50% of errors, *i.e.*, a median value,
4. CEP90—the radius of a circle encompassing 90% of errors, *i.e.*, ninth decile,
5. CEP95—the radius of a circle encompassing 95% of errors, *i.e.*, 95th percentile,
6. CEP99—the radius of a circle encompassing 99% of errors, i.e., 99th percentile,
7. Maximal error—the maximum value of an error recorded during a trial, denoted by max(*r_e_*)

## 6. Conclusions

**Figure 22.**
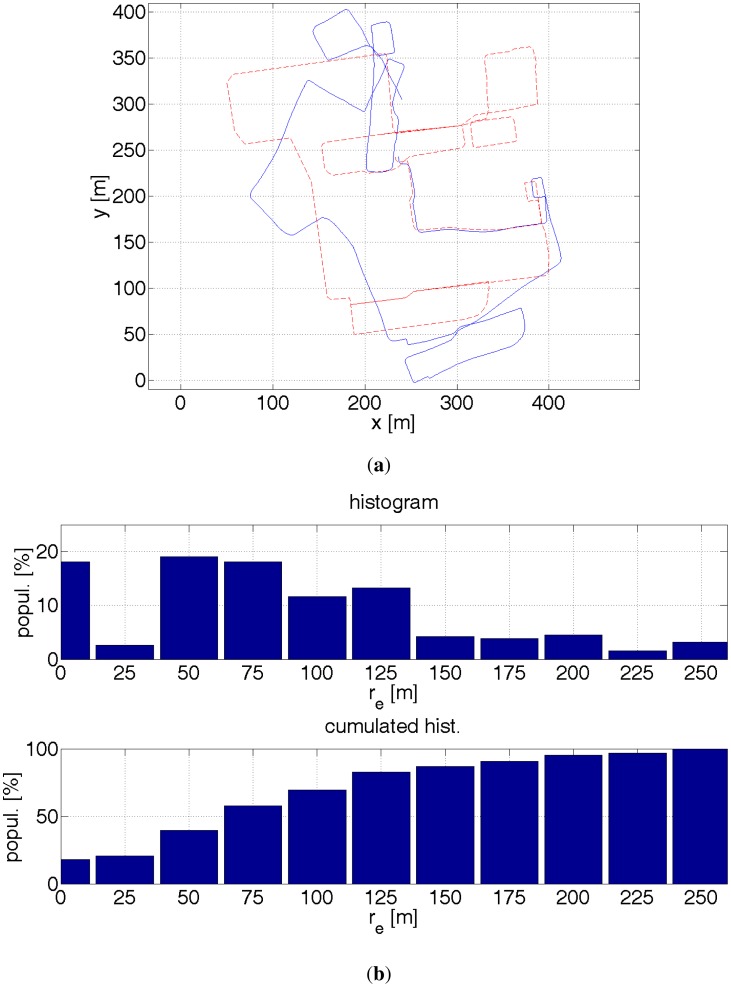
(**a**) Red dashed line—reference path. Blue solid line—estimated path with a help of the inertial sensors, *i.e.*, gyroscope for estimating direction change and accelerometers for counting steps and their lengths. As times goes on, the errors accumulate due to angular random walk. (**b**) Histograms of distance errors.

The results were calculated based on *ca.* 1,900 GPS readouts, 3,400 particle filter estimates and 19,000 pictures from the stereo camera. The particle filter using all available data sources provides by far the best accuracy. For 99% of the time the accuracy is better than 4.5 m. This is still too large an error for safe navigation of visually impaired pedestrians. The main source of errors were introduced by misestimated direction, which is visible in Figure 22 at coordinates (410 m, 125 m). The turn is misestimated by ≈20°. This error is too large for the non-linearity error mentioned in Section 3.1.1 or any other gyroscope error. Possibly the sensors came adrift and did not change its direction accordingly. This error, when not corrected immediately, has a ripple effect. This can be, however, taken care of. Also, more accurate sensors are available on the market. It is surmised that an error of 3 m for 99% of time is achievable.

It is interesting to note that when the initial position is known, the location of a pedestrian can be estimated without having to use a GPS receiver. The setup number 5 (see Table 1) gives best results after the setup number 4 and it is better than sole GPS readouts. Needless to say, this approach can be used in limited areas, e.g., inside buildings where GPS readouts are not available. The probability map concept limits the positioning error to the width of a street, *ca.* 10 m. Due to the imposed constraints on the pedestrian kinematics, the error dropped 3 times in terms of a root-mean-square error, which poses the best improvement from all other additional sources.

As mentioned before, on the bases of GPS readouts and the probability map, a user's location can be estimated within *ca.* 10 m. A stereo camera can refine the user's position down to 2 m, providing there are no unexpected objects, or difficult objects like trees, whose crown's radius could differ from the assumed 2 m. GPS readouts complement nicely with the algorithm in which the depth images derived from terrain model and stereoscopy are compared. In the presence of high buildings, GPS readouts are compromised. Under these circumstances the image matching algorithm performs well as the walls of buildings are confidently detectable by a stereo camera. On the other hand, in an open space GPS readouts are accurate down to, *ca.* 2–3 m. Then in turn, the image matching algorithm has no reference points to perform a viable comparison.

The presented algorithm works off-line. It is quite computationally demanding. The particle filtering algorithm requires generation of *ca.* 500 particles for good positioning accuracy. However, the task can be handled on-line without any optimization techniques by a dual core, 2.5 GHz standard notebook.

## Figures and Tables

**Figure 16.**
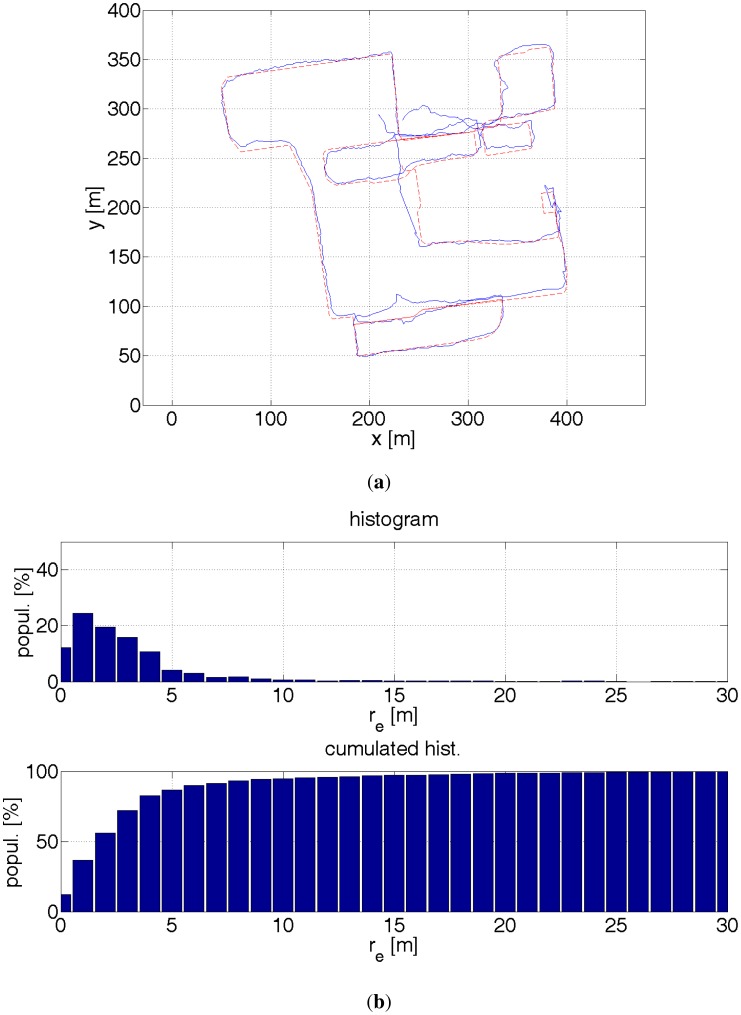
(**a**) Red dashed line—reference path. Blue solid line—estimated path with a help of a GPS receiver. In the presence of buildings, the GPS readouts are ≈30 m inaccurate. The errors depends also on the satellites configuration against a building. This can be noticed for coordinates (220 m, 80 m). The same place was revisited after a couple of minutes and the positioning error was very different. (**b**) Histograms of distance errors.

**Figure 17.**
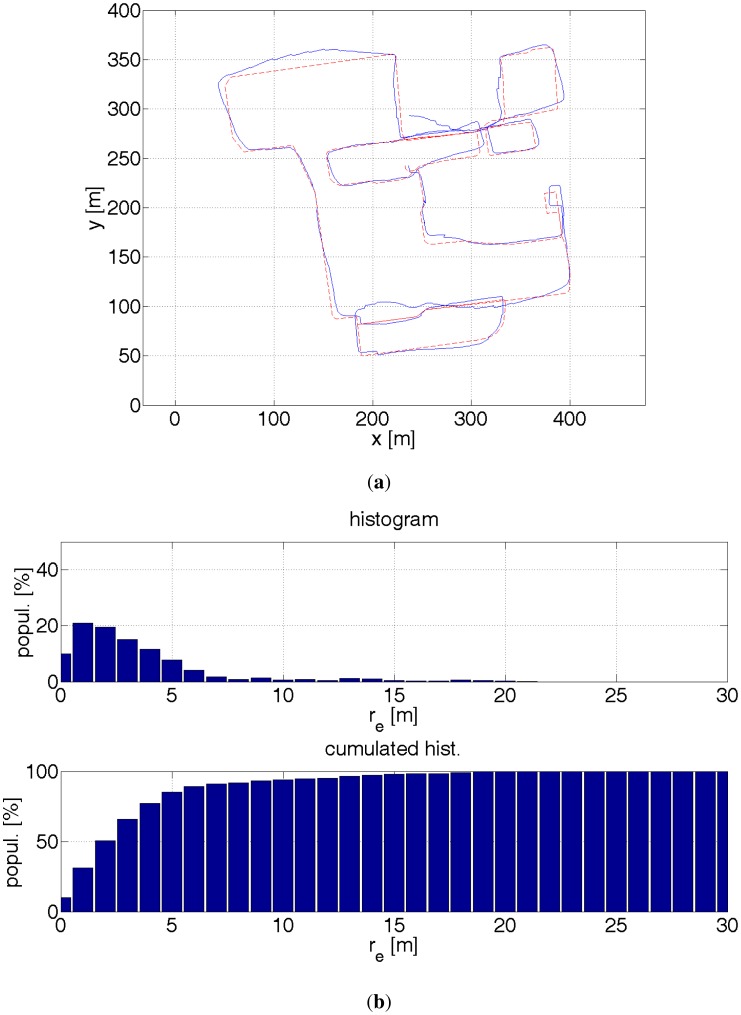
(**a**) Red dashed line—reference path. Blue solid line—estimated path with a help of GPS receiver and inertial sensors. The readouts from the inertial sensors enable to eliminate big errors. The accuracy improvement is not significant due to errors in estimating direction. (**b**) Histograms of distance errors.

**Figure 18.**
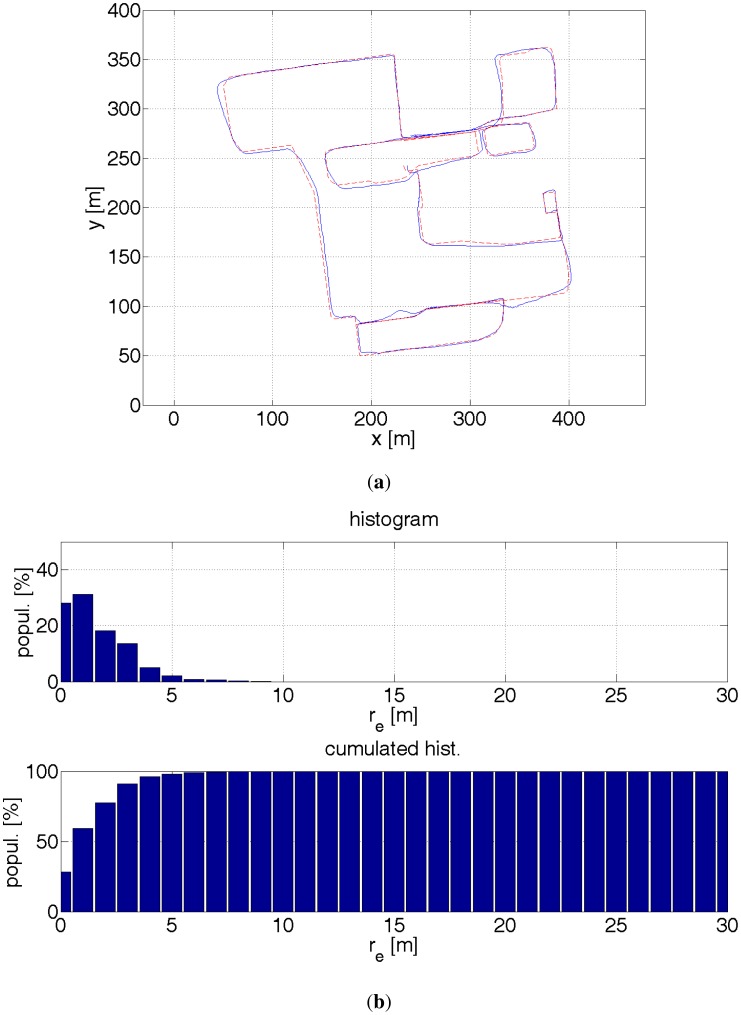
(**a**) Red dashed line—reference path. Blue solid line—estimated path with a help of GPS receiver, inertial sensors, probability map. The probability map eliminates direction errors, by pruning particles that diverge from a reference direction. Therefore errors are instantly eliminated and do not propagate. The positioning errors are then described by random walk with bounds. (**b**) Histograms of distance errors.

**Figure 19.**
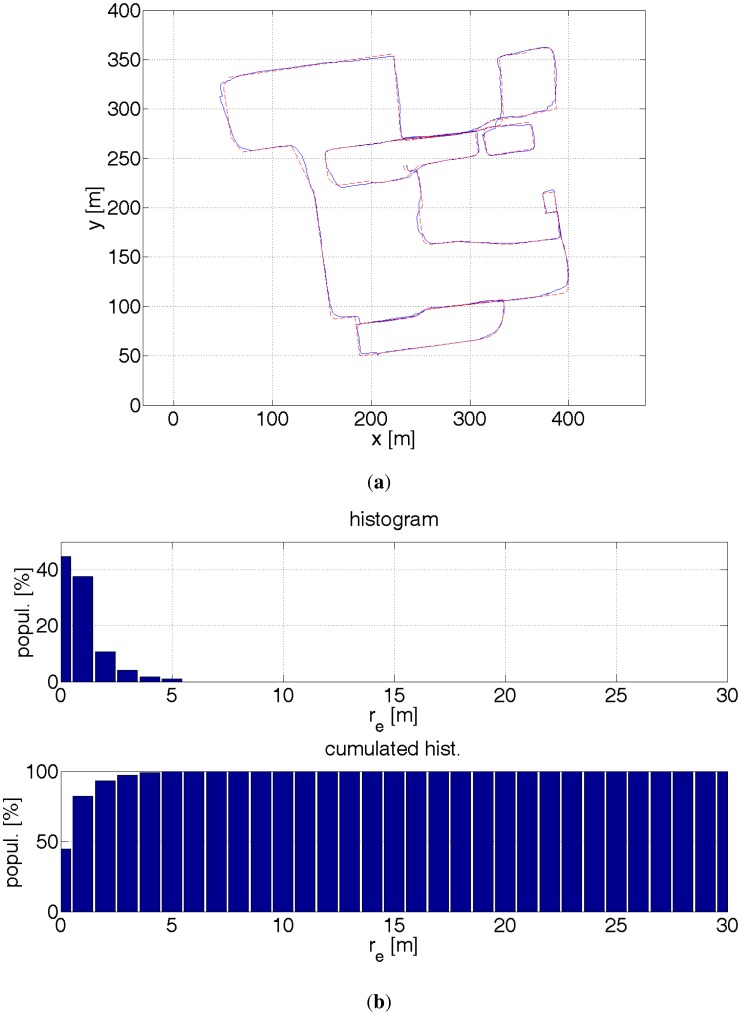
(**a**) Red dashed line—reference path. Blue solid line—estimated path with a help of GPS receiver, inertial sensors, probability map, stereo camera and 3D model of the environment. The positioning is quite precise. The errors are mainly introduced by the error in estimating the heading direction. (**b**) Histograms of distance errors.

**Figure 20.**
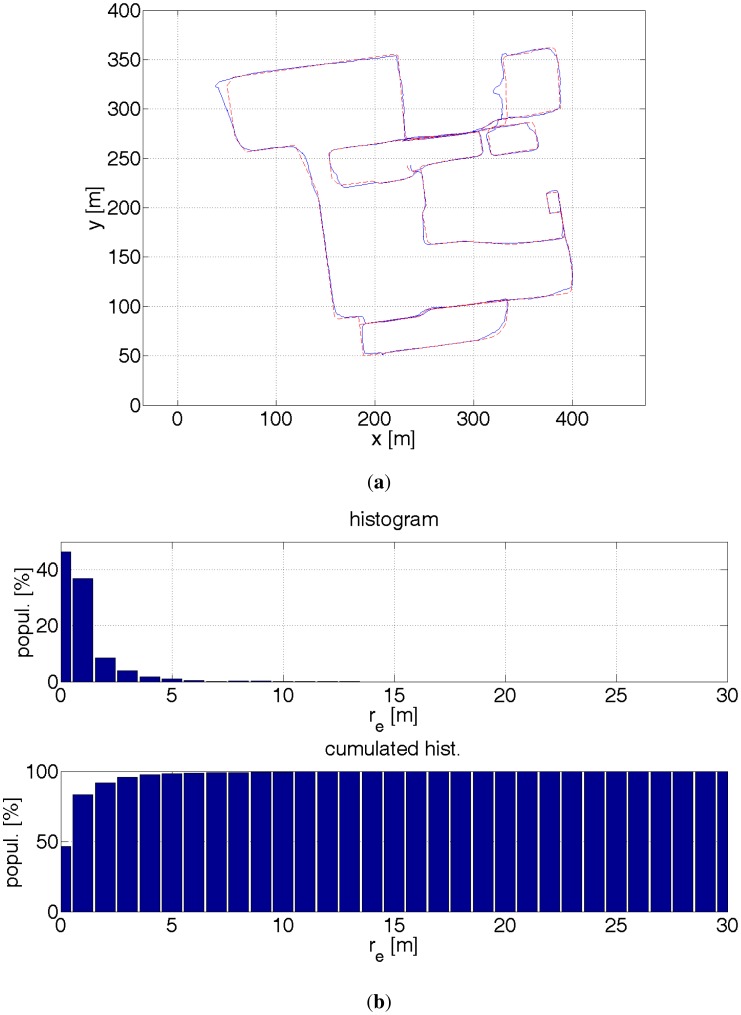
(**a**) Red dashed line—reference path. Blue solid line—estimated path with a help of inertial sensors, probability map, stereo camera and 3D model of the environment. When the initial position is known, the filter can estimate the pedestrian location with smaller errors than the GPS receiver. (**b**) Histograms of distance errors.

**Figure 21.**
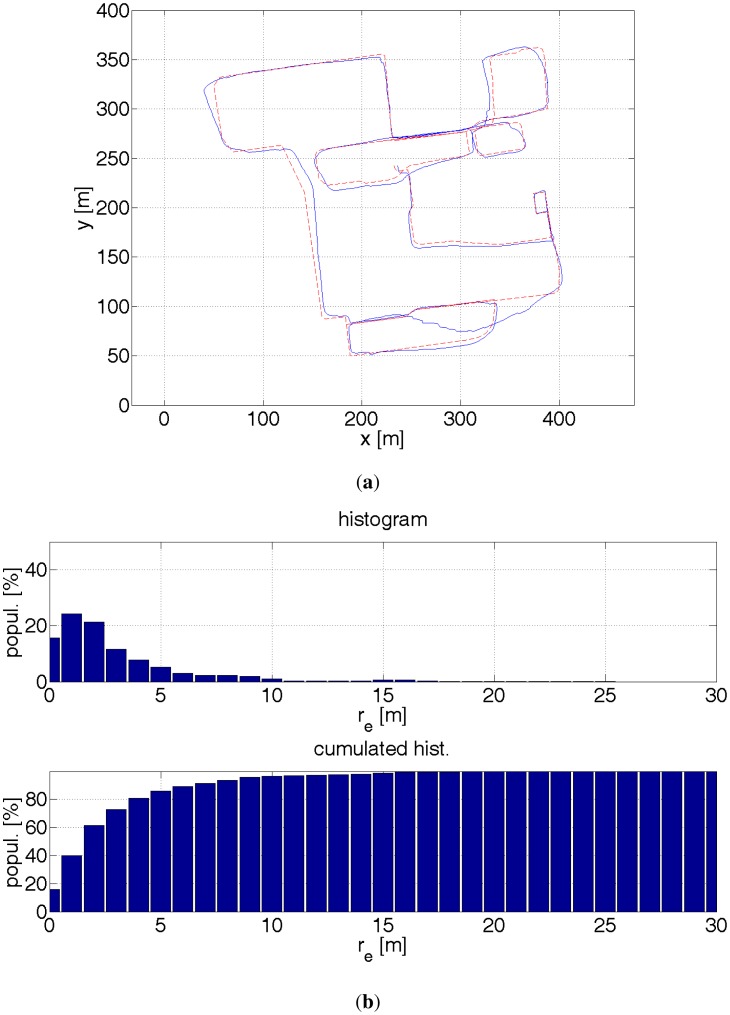
(**a**) Red dashed line—reference path. Blue solid line—estimated path with a help of GPS receiver, inertial sensors, probability map, stereo camera and 3D model of the environment. The error of estimating direction at (400 m, 120 m) introduces big errors later on. The filter managed to recover at (240 m, 80 m). (**b**) Histograms of distance errors.

**Table 1.** Results of the trial at the University Campus. Seven setups of data sources were simulated. The sign ‘+’ in a column denotes that a given data source was included in the simulation. Otherwise the data source is not used. Setup number 4 was carried out with all data sources on. The results for this setup are best.

Setup	1	2	3	4	5	6	7
GPS	+	+	+	+			
Inertialsensors		+	+	+	+	+	+
Probability map			+	+	+	+	
3D matching algorithm				+	+		
RMS*_e_* [m]	5.23	4.96	2.10	1.26	1.65	4.39	107.5
*μ_e_* [m]	3.27	3.45	1.56	0.87	0.95	2.94	86.4
CEP50 [m]	2.19	2.47	1.24	0.57	0.55	1.89	76.4
CEP90 [m]	6.54	6.79	3.39	2.03	2.13	6.92	182
CEP95 [m]	10.7	12.0	4.28	2.90	3.19	9.09	210
CEP99 [m]	23.3	18.2	6.41	4.50	7.02	16.0	261
max(*r_e_*) [m]	30.6	20.6	8.64	5.24	12.8	25.5	265
Figure	16	17	18	19	20	21	22
